# Diagnostic and prognostic role of liquid biopsy in non-small cell lung cancer: evaluation of circulating biomarkers

**DOI:** 10.37349/etat.2020.00020

**Published:** 2020-10-30

**Authors:** Giovanni Vicidomini, Roberto Cascone, Annalisa Carlucci, Alfonso Fiorelli, Marina Di Domenico, Mario Santini

**Affiliations:** 1 Department of Translation Medicine, Thoracic Surgery Unit, University of Campania “Luigi Vanvitelli”, 80131 Naples, Italy; 2 Department of Precision Medicine, University of Campania “Luigi Vanvitelli”, 80131 Naples, Italy; University of Campania “Luigi Vanvitelli”, Italy

**Keywords:** Liquid biopsy, lung cancer, circulating tumor cells, circulating tumor DNA, exosomes

## Abstract

Lung cancer is still one of the main causes of cancer-related death, together with prostate and colorectal cancers in males and breast and colorectal cancers in females. The prognosis for non-small cell lung cancer (NSCLC) is strictly dependent on feasibility of a complete surgical resection of the tumor at diagnosis. Since surgery is indicated only in early stages tumors, it is necessary to anticipate the timing of diagnosis in clinical practice. In the diagnostic and therapeutic pathway for NSCLC, sampling of neoplastic tissue is usually obtained using invasive methods that are not free from disadvantages and complications. A valid alternative to the standard biopsy is the liquid biopsy (LB), that is, the analysis of samples from peripheral blood, urine, and other biological fluids, with a simple and non-invasive collection. In particular, it is possible to detect in the blood different tumor derivatives, such as cell-free DNA (cfDNA) with its subtype circulating tumor DNA (ctDNA), cell-free RNA (cfRNA), and circulating tumor cells (CTCs). Plasma-based testing seems to have several advantages over tumor tissue biopsy; firstly, it reduces medical costs, risk of complications related to invasive procedures, and turnaround times; moreover, the analysis of genes alteration, such as *EGFR*, *ALK*, *ROS1*, and *BRAF* is faster and safer with this method, compared to tissue biopsy. Despite all these advantages, the evidences in literatures indicate that assays performed on liquid biopsies have a low sensitivity, making them unsuitable for screening in lung cancer at the current state. This is caused by lack of standardization in sampling and preparation of specimen and by the low concentration of biomarkers in the bloodstream. Instead, routinely use of LB should be preferred in revaluation of patients with advanced NSCLC resistant to chemotherapy, due to onset of new mutations.

## Introduction

Although in recent years we assisted in a decrease in the mortality rate from lung cancer, it is still one of the main causes of cancer-related death, together with prostate and colorectal cancers in males and breast and colorectal cancers in females. One of the main problems affecting mortality is an advanced stage of disease at the time of diagnosis, which translates into the inability to proceed with surgical treatment, significantly lowering the survival rate at 5 years [1]. Nevertheless, according to the National Cancer Institute, patients with metastatic stage-IV lung cancer have a 5-year survival rate of only 4.7% compared with a 5-year survival rate of up to 56.3% for those with stage-I cancer [2].

One of the causes of this modest result is the late diagnosis of lung cancer due to the late occurrence of specific cancer-related symptoms; therefore, more than two-thirds of all cases are detected in advanced and inoperable stages. Thus, the need for an early detection of non-small cell lung cancer (NSCLC) is essential to ensure the best chance of therapeutic success. During the past two decades, chemotherapy with platinum has been the best choice in all cases of advanced NSCLC. However, new targeted agents, such as tyrosine kinase inhibitors (TKIs), manifest greater efficacy than chemotherapy in patients with genomic mutations. Running a screening program by performing a low-dose CT scan (LDCT) has been shown to help lowering lung cancer mortality rate. Nevertheless, the routine usage of this method has several disadvantages, including the risk of tissue damage by radiation and the eventuality of obtaining false positives [3, 4]. The availability of a tool able to be coupled with the LDCT, such as the analysis of circulating tumor markers in plasma, gives us encouraging results for setting up a screening program that is as effective as possible [5]. The samples to be analyzed are obtained from peripheral blood, urine, and other biological fluids, making their collection simple and non-invasive.

**Figure 1.**
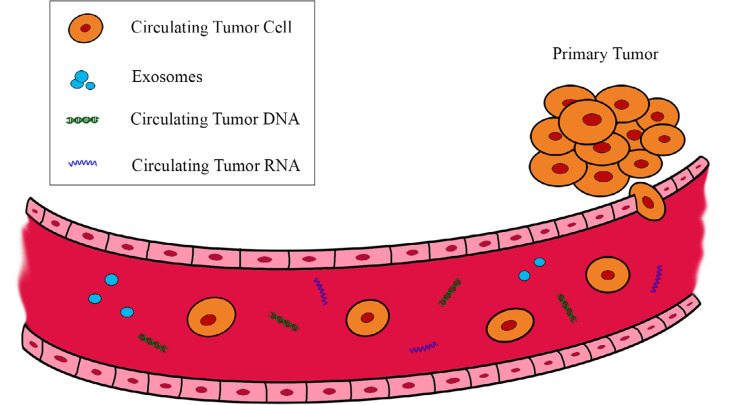
Main circulating biomarkers originating from the tumor

In the diagnostic and therapeutic pathway of NSCLC, neoplastic tissue samples are obtained using invasive methods, such as CT-guided fine needle aspiration biopsy (FNAB), flexible bronchoscopy (FBS) biopsies, or video-assisted thoracoscopic surgery (VATS), used for the definition of tumor histology and molecular investigations for guiding therapeutic choices. However, these methods are not free from disadvantages, being able to cause complications such as pneumothorax, haemothorax, spread of tumor, up to serious events such as pulmonary embolism [6]. For this reason, in the last decade, the prospect of obtaining neoplastic material quickly and with a minimally invasive approach has become increasingly concrete, as a valid alternative to the standard biopsy. Liquid biopsy (LB) represents a non-invasive approach to test tumor biomarkers in biological fluids. In particular, it is possible to detect in the blood different tumor molecules, such as cell-free DNA (cfDNA) with its subtype circulating tumor DNA (ctDNA), cell-free RNA (cfRNA), and circulating tumor cells (CTCs) [7] (Figure 1). The cfDNA can be commonly found in the peripheric blood, released from normal apoptotic cells, but it can also be produced by tumor cell necrosis. CTCs are cancer cells different from the normal cell population of the blood: they reach the vessels after detaching themselves from the tumor lesion and can be grouped in clusters or isolated. Finally, in the bloodstream, we can also find cfRNAs, released by cancer cells, often associated with exosomes that considerably increase their resistance to RNases [8]. Plasma-based testing has been mainly established for the analysis of *EGFR* hotspot mutations by quantitative real-time polymerase chain reaction (qRT-PCR) or digital PCR (dPCR). Next-generation sequencing (NGS) has also recently been developed to allow the analysis in plasma of several genes in parallel, with resolution down to 0.1% allele frequency [9] and this is crucial for the stratification of late-stage NSCLC patients at baseline in routine clinical care.

## Diagnostic and prognostic value of circulating biomarkers

The execution of LDCT alone, although characterized for its high sensitivity in the diagnosis of pulmonary nodules, is however burdened by a high number of false positives, often causing overdiagnosis. Since the prognosis of NSCLC is largely dependent on the possibility of performing a radical surgical resection, the need for an easily executable screening instrument with good sensitivity and specificity has emerged clearly.

An event that occurs early in the development of neoplastic pathogenesis is the release of CTCs from the main lesion or its metastases, that can occur in the bloodstream even when the tumor is less than 1 mm in size [4, 10, 11]. In literature, different system designed for CTCs analysis have been described with different costs and many advantages compared to tissue biopsy. A new micro-filter system to detect the CTCs with low costs was described by Cheng et al. [12], whereas Maheswaran et al. [13] demonstrated the ability to identify epidermal growth factor receptor mutations from CTCs isolated with a microchip device [14]. Actually, a recent study conducted at the MD Anderson Cancer Center (Houston, Texas), concluded that the NGS analysis on the tumor genome obtained from LB can completely replace tissue biopsy with the advantage of a reduced turnaround time (TAT). Moreover, plasma-based NGS is able not only to detect targetable genome but also changes in mutations from baseline to minimal disease progression thus having a prognostic role [9].

This study by Heeke et al. [9], shows NGS is a feasible system in routine clinical practice for detection of CTCs mutations, with a good correlation with NGS tissue results in 67% of patients. An interesting result is the ability of NGS system on LB to obtain an optimized detection of genomic alteration since the mutations identified from tissue DNA could be sometimes absent or non-detectable due to heterogeneity in tissue biopsies from the same patients.

Krebs et al. [10] firstly described the prognostic utility of CTCs, as detected by Cell-Search^®^ System in patients with an advanced NSCLC. A significant correlation is observed between CTC count and response to treatment. Basically, they suggest a cut-off value of > 5 CTCs (in 7.5 mL of blood) as highly significant in predicting worse prognosis compared with those patients with < 5 CTCs, independently from all other traditional prognostic factors (stage and performance status). Furthermore, CTCs play an important role in choosing the therapeutic approach; CTCs isolation could be useful for monitoring responses to therapy and predicting whether patients with NSCLC will benefit from treatment within a few weeks. A switch to an alternative therapeutic regimen can be necessary for all patients whose CTCs remain or become elevated after one cycle of standard chemotherapy. Indeed, a recent report demonstrated that radiological findings alone during the follow-up represent a poor predictor for overall survival in a pooled individual data analysis from 284 patients receiving treatment in phase II trials [15].

CTCs count in patients with comorbidities, such as chronic obstructive pulmonary disease (COPD), allows a diagnostic orientation from 1 to 4 years before the detection of a macroscopic lesion on CT-scan and the consequent surgical resection of the tumor, significantly improving the prognosis [16]. CTCs count could predict the presence of metastases; the cancer cells detaching from the main lesion are initially in a quiescent state, without proliferative activity. Once a suitable tissue for colonization has been reached, these cells return to a proliferative activity, producing metastases [17]. Therefore, the crucial point is to evaluate the replication activity of DNA, a sign of the ongoing proliferation of the cancer cell; An et al. [18] obtained promising results with the analysis of the Cell Division Cycle 6 (cdc6), a key factor for the beginning of DNA replication with proto-oncogene function. Although cdc6 was positive in only 20% of NSCLC patients recruited in the study, all positive subjects had metastases. However, the presence of CTCs at diagnosis in peripheral venous blood does not seem to be correlated independently with a worse prognosis.

The search for CTCs presents a higher diagnostic strength when associated with other biomarkers. A work by Zheng et al. [19] evaluated the combination of CTCs analysis (with a cut-off value of 12 units), carcinoembryonic antigen (CEA) levels, and CT-scan features in the diagnosis of the solitary pulmonary nodule. This association showed a sensitivity of 77.8% and a specificity of 90%, especially in upper lobe lung lesions, subsolid nodules, and nodules ≥ 8 mm in diameter.

CTCs were also combined with CCNI, EGFR, FGF19, and FRS2 expression levels obtained from salivary mRNA, in order to differentiate NSCLC patients from control healthy subjects. Using this combination of tests, Gu et al. [20] found both high sensitivity (92.1%) and specificity (92.9%).

One of the limitations of CTCs dosage is the low sensitivity of *in vitro* analyses, essentially due to the limited volumes of blood obtained from the peripheral sampling. A method that seems able to overcome this drawback is the CellCollector^®^, a probe coated with EpCAM antibodies inserted into a peripheral vein through a needle cannula and exposed for 30 min to the blood flow. This device is able to separate circulating epithelial cell adhesion molecule-positive cells thus allowing a higher specificity. Using this method, He et al. [21] reported a detection rate of 62.5% in a group of patients diagnosed with stage I and II NSCLC after surgical treatment, with no false positives in the control group consisting of healthy subjects.

The ctDNA is an additional useful biomarker that can be used for the early diagnosis of NSCLC and for the analysis of tumor genetic mutations [22, 23, 24]. There are several methods described for its analysis, such as the allele-specific PCR, the dPCR droplet (ddPCR), and the amplified refractory Mutation System (ARMS) PCR. However, these tests have the limit to be able to identify only a specific mutation and in a small part of the genes. Thanks to the introduction of NGS, we have now the opportunity to identify a wider spectrum of mutations by analyzing a single sample, compared to other methods [25, 26, 27].

Leung et al. [28] demonstrated the usefulness of ctDNA as a diagnostic test in patients with early stage NSCLC, analyzing the presence of *EGFR*, *KRAS*, and *p53* mutations, mostly frequent in adenocarcinoma and epidermoid lung carcinoma. When compared to the analysis of neoplastic tissue obtained with standard invasive procedures, this method showed comparable results, with a specificity of 89% in confirming the diagnosis. However, due to the low sensitivity, this test cannot be used to rule out a neoplastic disease. Co-amplification at lower denaturation temperature-PCR (COLD-PCR) for the amplification of the ctDNA sequences is able to identify an altered genetic locus but cannot discriminate the specific change in nucleotides.

A good alternative to the analysis of *EGFR*, *KRAS*, and *p53* mutations is the identification of methylations on the ctDNA; hypermethylation can, in fact, silence dozens of genes involved in the tumor suppression mechanism, favouring the neoplastic transformation of the cell. Chen et al. [29] applied this test to a group of patients with solitary pulmonary nodule < 3 cm in diameter, suspected for NSCLC. In patients with a nodule of 2.1 cm to 3 cm, the search for methylations on the *CDO1*, *SOX17*, and *HOXA7* genes showed a sensitivity of 91% and a specificity of 90%. In lesions with a diameter < 1 cm, however, the best combination of genes to be analyzed was shown to be *CDO1*, *TAC1*, and *SOX17*.

Circulating microRNA (miRNA) are nucleic acids that can be found in peripheral blood in subjects suffering from cancer. Their role in NSCLC pathogenesis seems to regulate the immune response, inhibiting the expression of programmed death-ligand 1 (PD-L1) and thus, promoting the mechanism of evasion of the tumor from the immune system. Unlike other types of RNA, such as messenger RNA, non-coding RNA, or circular RNA, miRNAs have a relatively longer half-life and represent an important biomarker in the diagnosis of NSCLC [30, 31]. The most frequently used tool for the quantification of miRNAs is the RNA isolation-qRT-PCR workflow which has the limit of not discriminating between miRNAs released by the tumor and those produced by normal cells, undermining the sensitivity of the test. Liu et al. [32] have proposed the use of a tethered cationic lipoplex nanoparticles (tCLN) biochip capable of selectively identifying RNA derived from tumor exosomes, allowing to discriminate subjects with NSCLC from healthy ones, with a sensitivity of 96.9% and a specificity of 93.3%.

A study of Fehlmann et al. [31] highlighted the possibility of looking for genome-wide miRNA profiles in the blood, in order to achieve an early diagnosis in asymptomatic patients. Blood samples from 3, 046 individuals were analyzed to match the results between patients with lung cancer, non tumor lung diseases (i.e. chronic obstructive pulmonary disease), extrapulmonary diseases, and healthy controls. This study showed the utility of miRNA signatures in whole blood samples for an early diagnosis of NSCLC. The minimal median expression value among the one hundred most statistically significant miRNAs was observed for 47 miRNAs in the lung cancer group, 25 miRNAs in nontumor lung diseases, 15 miRNAs in diseases not affecting the lung, and 13 miRNAs in unaffected control participants. These findings suggest a statistically significant up-regulation of *hsa-miR-17-3p* in patients with non-tumoral lung lesions but not in patients with lung cancer, as well as a down-regulation of *hsa-miR-140-5p* only in patients with lung cancer and a down regulation of *hsa-miR-628-3p* and *hsa-miR-374c-5p* in those with non-tumor lung diseases.

Therefore, this study recommends the circulating biomarker test as a future standardized approach that could be used in addition to imaging, sputum cytology, and biopsy tests.

Extra-cellular vescicles (EVs) are small membranous vesicles that are secreted by either normal or tumor cells. They are classified into three subclasses: exosomes, macrovesicles, and apoptotic bodies. Cancer cells have the ability to produce exosomes, a significant class of EVs with a spherical nano-sized architecture and a diameter of 40–100 nm and a density of 1.13–1.19 g/mL, and to release them into the bloodstream. These nano-sized vesicles contain many molecules including nuclear acids (e.g., double-stranded DNA and various subtypes of RNA), proteins, and lipids. They have an important role in maintaining homeostasis and they are carried by endosomal sorting complexes required for transport (ESCRT); in tumor cells, ESCRT is significantly altered and may cause the molecular profile inside exosomes to be largely modified so, tumor cell-derived exosomes are related to tumor progression.

Since exosomes can be easily acquired from most body fluids and characterized, they may serve as a promising LB bio markers of lung cancer. Ultracentrifugation-based technologies as well as commercially available kits are methods that are most commonly used for exosome extraction [33]. Exosomes have good longevity in the blood and can be effectively used as biomarkers for NSCLC since the genetic material composed of DNA and RNA transported inside them remains intact [34, 35, 36]. Among all the exosomes material, miRNAs showed the best results in the diagnostic and therapeutic pathway of NSCLC.

A study by Zhou et al. [37] identified a six-miRNA panel (miR-19b-3p, miR-21-5p, miR-221-3p, miR-409-3p, miR-425-5p, and miR-584-5p) which can be used for the diagnosis of pulmonary adenocarcinoma. Furthermore, miRNAs contained in the exosomes have been used for the differential diagnosis between pneumonic process and lung cancer, using pleural effusion as the basis of the LB [38, 39, 40].

The analysis of exosomal miRNAs is also useful for the prognostic evaluation of NSCLC, resulting significantly up-regulated in patients who have a recurrence of neoplastic disease compared to healthy subjects: they are also correlated to an advanced stage of the tumor [41].

In addition to nucleic acids, promising results for the early detection of NSCLC have been obtained by the dosage of serum proteins. Boccellino et al. [42] compared the serum obtained from 20 NSCLC patients with that of 10 healthy subjects, identifying α2-macroglobulin, α-microglobulin/bikunin and SERPINA1 as potential biomarkers for early diagnosis and surveillance in lung cancer. An increased bikunin on proteomic analysis seems to be related to recurrence of cancer, while SERPINA1 levels seem to decrease in the case of advanced lung cancer.

Finally, the soluble major histocompatibility complex class I polypeptide related sequence A (sMICA) obtained from the serum of lung cancer patients should be mentioned. Its role in the surveillance of lung cancer seems promising since high serum sMICA levels have been shown to be related to an advanced stage, poor differentiation, and, therefore, poor prognosis [43].

## Role of circulating tumor markers in therapy

New strategies that target survival pathways and interfere with proliferation of neoplastic cells, providing efficient and effective results, are being considered. The most important of these drugs are the TKIs, they interfere with tyrosine kinases, enzymes responsible for signal transduction in cancerogenesis.

Unfortunately, with progression of neoplastic disease, the pool of tumor cells may develop mutations that make TKIs ineffective, because they do not recognize their target anymore.

**Figure 2.**
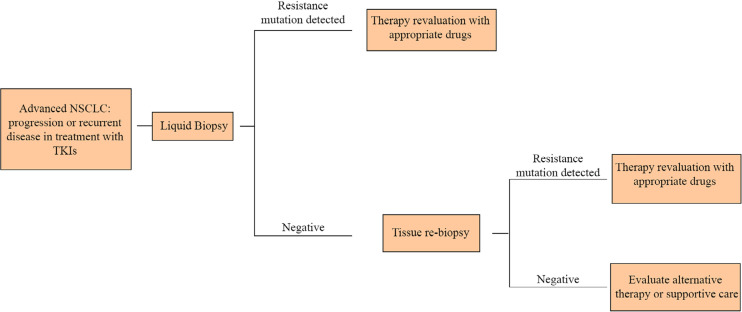
In the NSCLC therapeutic algorithm, liquid biopsy allows to cut down the invasive biopsies to obtain further tumor samples

Thus, in patients no longer responsive to TKIs, it is crucial to iterate analyses on tumor genome, in order to clarify the new mutation and set up a tailored therapy. In this scenario, the execution of repeated tissue biopsies could be not well tolerated by patients or it can also be too difficult to perform. LB overcomes these obstacles thanks to its easy execution, offering a quick and minimally invasive alternative to surgical biopsies of tumor samples (Figure 2).

*EGFR* mutation in lung cancer cells, especially in the case of adenocarcinoma, is correlated with an increased tumor growth and cancer progression. These *EGFR* mutations are mostly exon 19 deletions and L858R point mutation on exon 21 [44]. Patients with these mutations are treated with targeted therapy (EGFR-TKIs, such as erlotinib, gefitinib, afatinib); thus, detecting genomic alterations becomes crucial in order to select the appropriate treatment and develop further targeted agents.

Another common mutation is T790M in exon 20; it consists of an acquired resistance to EGFR-TKI therapy, bringing to a cancer progression. According to the Ontario Health (Quality) report [45], tissue biopsy is an imperfect gold standard in research for these types of mutations. Due to tumor heterogeneity, the *EGFR* T790M mutation might not be found in all tumor sites, so it is not totally helpful in case of disease progression. Furthermore, tissue biopsies require the use of interventional radiology to retrieve samples from internal organs with associated risk of pneumothorax, bleeding, pain, and discomfort. Detection of *EGFR* mutation in plasma-derived ctDNA from patients with lung cancers represents an interesting alternative to *EGFR* genetic analyses in patients that cannot undergo tissue biopsy due to their advanced disease and/ or poor performance status, and in cases of insufficient material obtained from tissue biopsies. A recent study of Sabari et al. [46] compared ctDNA and tissue DNA sequencing; the authors found a concordance between plasma and tissue-based sequencing, promoting a potential incorporation of this test into practice guidelines. They also support the idea that plasma ctDNA analysis with NGS matches patients to targeted therapy with a shorter test TAT compared with tissue NGS; thus, a driver alteration identified by plasma NGS can immediately direct clinical care. However, a negative result requires further investigation.

More recently, EGFR mutation tests that use cell-free ctDNA through an LB via plasma have been developed as an alternative or complementary test to tissue biopsy. Only a blood sample is needed for testing and this proves that LB is less invasive than tissue biopsy.

Papadimitrakopoulou et al. [22] state the possibility to find *EGFR* mutations, including the acquired T790M resistance mutation, in plasma ctDNA samples from patients with NSCLC. They found that plasma *EGFR* T790M detection was associated with a larger median baseline tumor size and presence of extrathoracic disease. Interestingly, the T790M resistance mutation seems to be more easily detected in the plasma of patients with metastatic disease *versus* locally advanced disease. This finding seems to be due to an adequate quantity of *EGFR* gene copies in ctDNA which correlates with the disease burden. As well, in this paper, the authors evaluate the clinical response to osimertinib, a third generation TKI specific also for T790M positive patients, and platinum-pemetrexed in patients with *EGFR* T790M-positive advanced NSCLC, according to the T790M status collected at the baseline from circulating tumor DNA (ctDNA). They proved that patients undergone osimertinib therapy had a better outcome than platinum-pemetrexed treated ones. Moreover, in patients with tissue T790M-positive NSCLC the absence of plasma T790M at the baseline is associated with longer progression-free survival, which may be attributed to a lower disease burden.

This finding is supported by results from another recent study [47] in which patients receiving osimertinib (*n* = 38) with low levels of T790M in ctDNA had improved progression free and overall survival in comparison with patients with higher levels; low levels of T790M and complete clearance after 2 months were also associated with better outcomes.

Tumor educated platelets are blood cells which house genetic material from cancer through interaction with tumor cells; for this reason, they can be used as biomarkers in the diagnosis of NSCLC. In addition, their identification is also useful in the course of therapy for the choice of the most effective drugs. We know that the two most important mutations for therapeutic purpose in pulmonary adenocarcinoma regard *EGFR* and anaplastic lymphoma kinase (*ALK*); the latter is found in 4% of cases and is associated with high therapeutic response to ALK TKIs crizotinib. Nilsson et al. [48, 49] identified the *EML4-ALK* rearrangement, which represents the most frequent mutation in lung adenocarcinoma, using reverse transcription-PCR in platelets. They found a sensitivity of 65% and a specificity of 100%. Due to the early development of tumor resistance to crizotinib, it is important to identify any early resistance mutations in NSCLC; in this setting, the use of tumor educated platelets is more reliable than an additional tissue biopsy [50, 51, 52]. In contrast to *EGFR*, for which substantial data exists, *ALK* rearrangement assessment in non-treated patients is limited due to need of enough pathological material of assessment of *ALK* status, and no prospective cohorts have been evaluated to date to compare *ALK* detection on LB *versus* tissue.

## Conclusion

Lung cancer patients benefit from the use of LB strategies to detect genetic mutations.

NGS, qRT-PCR and dPCR are newly tests introduced for that purpose.

These tests allow the collection of CTCs, ctDNA, miRNA, exosomes, and peptides used for the accurate characterization of tumor genome.

Many studies have demonstrated the usefulness of LB tests and their validity in addition or alternative to those cases in which the tissue biopsy was not feasible or repeatable; besides the advantage in terms of feasibility, it was widely proved to overcome the limit of tumor heterogeneity, not assessable when a tissue biopsy is performed.

One of the limits of these tests is the concentration of biomarkers in plasma; they depend on their releasing from tumor cells and can be sometimes too low to be detected. In patients with NSCLC with a squamous histology, for example, there is a fewer CTCs release in plasma than other histologies and this leads to poor sensitivity of the tests performed. Thus, a chance of false negatives must be considered.

The absence of a genomic alteration in one of these tests could be explained by the sample quality and the test sensitivity; so, more research is required to better understand the kinetics of tumor shedding and the effects of treatment (surgery, radiation, chemotherapy, and immunotherapy) on plasma levels of biomarkers.

Plasma is the most used sample but other biological fluids should not be ignored; in fact, tumor biomarkers could be detected even in sputum, urine, or pleural effusion, all liquid that can be easily collected. A combination of these different approaches may mitigate their low sensitivity. Another limit is the lack of standardization of samples preparation among various centers. This could lead to heterogeneous results and difficult data comparison from different institutions.

Plasma-based testing seems to have several advantages over tumor tissue biopsy; firstly, it reduces medical costs, risk of complications related to invasive procedures, and TATs; moreover, the analysis of mutations in genes, such as *EGFR*, *ALK*, *ROS1*, and *BRAF* safer with this method, compared to tissue biopsy. However, thanks to a faster TAT and the ability of these tests to assess the systemic genomic landscape and incorporate tumor heterogeneity compared to a tissue biopsy, plasma-based tests should be considered in patients with multiple metastatic sites or in a setting of acquired resistance to targeted therapies.

It was demonstrated that detection of ctDNA in plasma is an additional useful biomarker that can be used both for an earlier diagnosis of NSCLC and the analysis of tumor genetic mutations in all cases of acquired resistance. Thanks to the introduction of NGS, we have now the opportunity to identify a wider spectrum of mutations by analyzing a single sample, compared to other methods such as the allele-specific PCR, the ddPCR and the ARMS PCR, because they have the limit to be able to identify only a specific mutation and in a small part of the genes.

Additionally, plasma-based NGS is able to not only detect targetable mutations at baseline but also minimal residual disease at progression and it might consequently be beneficial as a prognostic tool.

Likewise, considering testing-related costs and effects, LB was more effective and less costly than tissue biopsy used alone. Using LB as a triage test led to the greatest number of correct treatment decisions (i.e. where people who were *EGFR* T790M positive received osimertinib and people who were *EGFR* T790M negative received chemotherapy). When considering all long-term costs and effects, LB was not cost-effective because of the high cost of the third-generation EGFR-TKI treatment (i.e. osimertinib). Thus, evaluation of costs is one of the unmet objectives until now.

Although relevant data concerning the therapeutic value of LB have already been obtained, further studies are needed to evaluate its role in early diagnosis of lung cancer and biological factor such as tumor shedding and ctDNA kinetics remain an ongoing area of investigation in the field of LB.

## Abbreviations

ALK: anaplastic lymphoma kinase
COLD-PCR: co-amplification at lower denaturation temperature-polymerase chain reaction
CT: computed tompography
CTC: circulating tumor cell
ctDNA: circulating tumor DNA
dPCR: digital polymerase chain reaction
EGFR: epidermal growth factor receptor
LB: liquid biopsy
LDCT: low-dose computed tomography
miRNA: microRNA
NGS: next-generation sequencing
NSCLC: non-small cell lung cancer
PD-L1: programmed death-ligand 1
qRT-PCR: quantitative real-time polymerase chain reaction
TAT: turnaround time
TKI: tyrosine kinase inhibitor

## Declarations

### Copyright

© The Author(s) 2020.
